# Tomographic Collection of Block-Based Sparse STEM Images: Practical Implementation and Impact on the Quality of the 3D Reconstructed Volume

**DOI:** 10.3390/ma12142281

**Published:** 2019-07-16

**Authors:** Sylvain Trépout

**Affiliations:** Institut Curie, Inserm U1196, CNRS UMR 9187, Université Paris Sud, Centre Universitaire, Bât. 110-112, 91405 Orsay CEDEX, France; sylvain.trepout@curie.fr; Tel.: +33-169-86-30-81

**Keywords:** scanning transmission electron microscopy (STEM), electron tomography (ET), sparse imaging, inpainting reconstruction, biological samples, *Trypanosoma brucei*

## Abstract

The reduction of the electron dose in electron tomography of biological samples is of high significance to diminish radiation damages. Simulations have shown that sparse data collection can perform efficient electron dose reduction. Frameworks based on compressive-sensing or inpainting algorithms have been proposed to accurately reconstruct missing information in sparse data. The present work proposes a practical implementation to perform tomographic collection of block-based sparse images in scanning transmission electron microscopy. The method has been applied on sections of chemically-fixed and resin-embedded *Trypanosoma brucei* cells. There are 3D reconstructions obtained from various amounts of downsampling, which are compared and eventually the limits of electron dose reduction using this method are explored.

## 1. Introduction

Accessing the ultrastructure and cellular organization of cell components has always been of great help in deciphering their functions and mechanisms inside the cell [1,2,3,4]. Electron tomography (ET) is a powerful tool to seize the global overview of cellular architecture in 3D at the nanometer scale. However, transmission electron microscopy (TEM)-based methods are limited to the study of thin samples because the substantial electron scattering occurring in thick samples leads to the collection of images with weak contrast and poor signal-to-noise ratio (SNR). Even though more detailed and contrasted images can be obtained by filtering out inelastically scattered electrons using energy filters, above about 300 nm (or little more depending on the acceleration voltage of the microscope) TEM-based methods are inefficient to capture quality images. Other methods have been developed to image thick specimens. Focused-ion beam (FIB) milling [5,6,7] only reduces the thickness of thick samples so that they become thin enough, but do not allow to image thick samples. Serial sectioning aims at imaging thick samples but it is a tedious task [8]. An alternative to TEM, is the use of scanning transmission electron microscopy (STEM) that is based on the raster scanning of an electron beam focused on the sample, the transmitted electrons being collected by one (or more) post-specimen detector(s) [9,10]. Thanks to the beam geometry; through the point-to-point imaging pattern and the absence of electromagnetic lens post-specimen, it has been shown that STEM is able to produce high SNR and high contrast images of thick biological samples [11,12]. As opposed to TEM, STEM is an incoherent imaging mode, less affected by the strong scattering of the electron waves occurring in thick samples. Importantly it has been shown that bright-field imaging produces images of higher fidelity compared to annular dark-field because of the multiple electron scattering that can occur inside the sample [13,14]. It has also been shown on cryo-preserved prokaryotic cells that at equivalent electron doses, the use of STEM generates higher SNR images compared to TEM while reducing structural damages [15]. More recently, simulations have shown that biological samples of micron thicknesses (“and beyond”) could be studied in cryo-STEM tomography [16]. However, studying thick specimens involves the use of high electron doses to maintain a sufficient number of electrons at the detector level, increasing radiation damages. Strategies to reduce the electron dose are of main significance to preserve the sample integrity.

In ET, several strategies based on sparse acquisition (i.e., downsampling) of the data have been proposed to reduce radiation damages. Restoration of missing information is performed using data compression approaches based on algorithms such as compressive-sensing (CS) or inpainting. In previous work, several algorithms such as discrete algebraic reconstruction technique [17], total variation minimization [18], CS [19,20,21] have been developed. In these different works, the downsampling is performed either by (i) reducing the number of acquired tilt angles or by (ii) reducing the number of pixels collected per image (i.e., sparse images). Very recently, the limits of the reduction of collected tilt-angles associated with sparsity-exploiting reconstructions have been explored and show that they are highly dependent on the sample properties [22]. More precisely, specimen complexity and noise severely degrade reconstruction quality in sparsity-exploiting methods. Biological samples are beam sensitive complex systems requiring the use of a low electron dose, hence the collection of noisy images. For these reasons, strategies reducing the number of collected tilt-angles perform very well on samples with low complexity, whereas they do not perform so well on biological samples because of the very intricate state of beam-sensitive biological matter. Whereas, it is trivial to reduce the number of acquired tilt angles in TEM or STEM tomographic experiments, the actual implementation of sparse imaging at the level of the image itself is much more complex. In TEM, there is no strategy yet to collect sparse images whereas STEM has the potential to collect sparse images thanks to its point-to-point imaging pattern as shown for example in a STEM-Electron Energy Loss Spectroscopy experiment [23]. Despite the possibility to collect sparse images in STEM, most of the works studying sparsity-exploiting strategies simulate sparse images in silico post-acquisition [19,21,24,25]. In rare experiments, sparse images were experimentally collected, the electron beam being either blanked using a fast electromagnetic shutter at the level of the condenser [26] or being driven using a dedicated scan system [27,28]. Very recently, Vanrompay et al. used a fast electromagnetic shutter to perform sparse imaging in 3D on gold nanoparticles [29].

There is a growing interest in biology to use sparse acquisitions in ET experiments [30,31] and the need to develop such acquisition methods is urgent. The goal of the present work is to develop a tomographic acquisition method able to collect sparse images to study beam-sensitive thick biological samples in STEM. Section 2 describes the materials used and the electron microscope setup. Section 3.1 presents first the practical implementation of sparse images in STEM on 2D images. Section 3.2 extends the method to perform sparse imaging during a tilt-series to perform 3D imaging. In Section 3.3 the method is applied on a 500 nm-thick chemically-fixed and resin-embedded *Trypanosoma brucei* sample. The pros and cons of sparse imaging using the scanning coils of the electron microscope and the effect of the electron dose reduction on the quality of the 3D reconstruction are discussed thereafter.

## 2. Materials and Methods

### 2.1. Test Samples

The validation of the practical implementation to collect 2D sparse images has been performed on a carbon crossed line grating replica grid and on iron oxyde nanoparticles [32]. The carbon replica used in this work had evaporated Au/Pd atoms and latex spheres at the surface (EMS #80055). The development and the validation of the 3D tomography workflow was performed on the carbon crossed line grating replica grid.

After validation of the tomography workflow, it was applied on *T. brucei* resin Sections. *T. brucei* cells (strain 427) were cultured in SDM79 medium supplemented with hemin and 10% fetal calf serum [33]. Cells were chemically fixed directly in the culture medium with 2.5% glutaraldehyde for 30 min, exhaustively rinsed in phosphate-buffered saline, post-fixed in 2% OsO4 for 30 min at 4 °C in the dark, dehydrated in baths of increasing ethanol concentrations, and embedded in Epon as previously described [34]. After resin polymerization at 60 °C for 48 h, resin blocks were cut to produce sections of 500 nm which were deposited on bare 200 mesh copper grids (EMS #G200-Cu).

### 2.2. Experimental Setup of the Electron Microscope

The 200 kV field emission gun electron microscope (2200FS, JEOL, Tokyo, Japan) in scanning mode was set up to enable imaging conditions for thick specimens using the following parameters (condenser lens aperture: 40 µm, camera length: 80 cm, semi-convergence angle was 9.3 mrad and outer bright-field semi-collection angle was 5 mrad). Images collected between 20,000 × and 30,000 × magnifications (corresponding pixel sizes were 2.08 and 1.38 nm respectively) were digitized using a Digiscan II ADC module (16 bits). The beam current was 2.2 pA.

### 2.3. Tilt-Series Collection, Alignment and Reconstruction

Tilt-series on the crossed line grating replica grid were collected at 30,000 × magnification, between −65° and +63° using 0.5° tilt increments and the dwell time was set to 8 µs. Using these collection conditions, the final electron dose received by the sample after completion of the tilt-series has been estimated around 1000 e^−^/Å^2^ for fully collected images. These settings have been used to generate high contrast images. During the tilt-series, (i) a sparse image downsampled to 12.5% of the total amount of pixels and (ii) a fully collected image were collected at each tilt angle. Fully collected images were used as ground truth images. Tilt-series on the *T. brucei* resin section were collected at 20,000 × magnification, between −67° and +68° using 1° tilt increments and the dwell time was set to 1 µs. These settings correspond to a significant electron dose reduction compared to the settings used on the crossed line grating test sample. The electron dose was deliberately diminished to match very low dose collection conditions. Using these collection conditions, the final electron dose received by the sample after completion of the tilt-series has been estimated around 70 e^−^/Å^2^ for fully collected images. To study the impact of the electron dose reduction on the quality of the reconstruction, a total of eight sparse images were collected for each tilt angle. The downsampling amount in the sparse images ranged between 3.125% and 25%.

After collection of the whole tilt-series, block-based sparse images were reconstructed in Matlab using 250 iterations of the inpainting algorithm developed by Garcia [35,36]. Fewer iterations did not give satisfactory results whereas more iterations seemed unnecessary and cost longer computing times. Tilt-series alignment was performed using cross-correlation in Etomo [37,38] and particular care was taken to produce the best aligned tilt-series possible by playing with alignment parameters in Etomo such as filtering and trimming of the images. Reconstructions were generated by weighted-back projection (WBP) in Etomo.

### 2.4. Reconstruction Quality Assessment

The quality of reconstructions was initially assessed by visual inspection. However, to better characterize the reconstructions, image quality descriptors (IQD) were also measured on individual Z-slices of the 3D volumes. Entropy (*H*), root mean square contrast (*C_rms_*) and Michelson’s contrast (*C_m_*) were computed using the following equations [39]:

$$H=-\sum_{i=1}^{n}P_{i}\log_{2}P_{i}$$

*H* is the entropy of the element containing *n* pixels and *Pi* is the appearance probability of the pixel value *i* in the element.

$$C_{rms}=\sqrt{\frac{1}{MN}\sum_{i=1}^{M}\sum_{j=1}^{N}{\left(I_{xy}-\bar{I}\right)}^{2}}$$

*C_rms_* is the root mean square contrast, *M* and *N* are the dimensions of the data in *x* and *y* respectively, $I_{xy}$ is the value of the pixel at position *xy* and $\bar{I}$ is the mean pixel value.

$$C_{m}=\frac{I_{\max}-I_{\min}}{I_{\max}+I_{\min}}$$

*C_m_* represents the Michelson’s contrast, $I_{\max}$ is the maximum pixel value and $I_{\min}$ is the minimum pixel value.

In ET, the Z-dimension of a reconstruction is large enough so that the entirety of the object of interest can fit in the 3D volume. The quality of the reconstructions has been estimated by computing the ratio between IQD values measured at the level of the object of interest and IQD values measured above and below where there should be no object (later on referred as the background). In an ideal missing-wedge-free and noise-free 3D reconstruction, Z-slices located above and below the object of interest should have no contrast. In a 3D reconstruction based on experimental images that contain noise, the same Z-slices suffer from missing-wedge and noise reconstruction artifacts.

### 2.5. Data Presentation

Images presented in this work have been generated using ImageJ (v1.51j8) [40]. Artworks have been designed in Blender. The computation of IQD and corresponding figures were performed in Matlab. IQD ratios data were fitted to the following model function using non-linear least-squares regression:

f(x)=A−1/(B+Cx)

## 3. Results

### 3.1. Practical Implementation in 2D

#### 3.1.1. Collection of Sparse STEM Images

**Figure 1:**
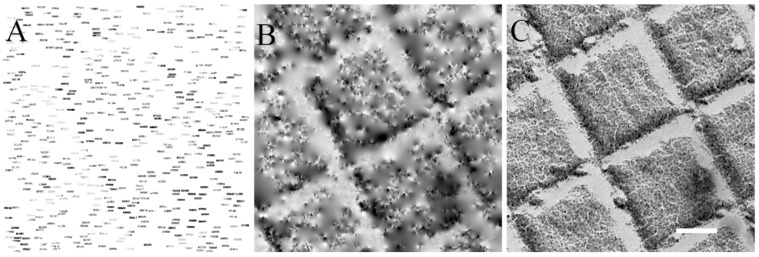
Collection of a sparse scanning transmission electron microscopy (STEM) image, inpainting reconstruction and comparison with ground truth. (**A**) Sparse STEM image downsampled to 12.5% using 512 blocks of 16 × 4 pixels. The white background corresponds to uncollected pixels. (**B**) Sparse image A after inpainting reconstruction. (**C**) Ground truth fully-collected STEM image of the very same ROI on the grid. Scale bar is 200 nm. The three subfigures have the same scale.

Collection of non-overlapping pixels blocks randomly distributed over the area of interest (ROI) was performed using an in-house developed Digital Micrograph (DM) script. The Digiscan II software of DM has built-in functions that allow the collection of pixel blocks which dimensions can be 16 × 4 pixels. This collection scheme was used on a carbon crossed line grating grid to collect 12.5% of the pixels (Figure 1A). The white background represents uncollected pixels. Inpainting was then computed to recover missing pixels (Figure 1B). The pattern of the crossed line grating replica becomes apparent after the inpainting reconstruction and resembles the ground-truth image of the same ROI (Figure 1C).

#### 3.1.2. Accuracy of the Electron Beam Positioning during Sparse Imaging

**Figure 2:**
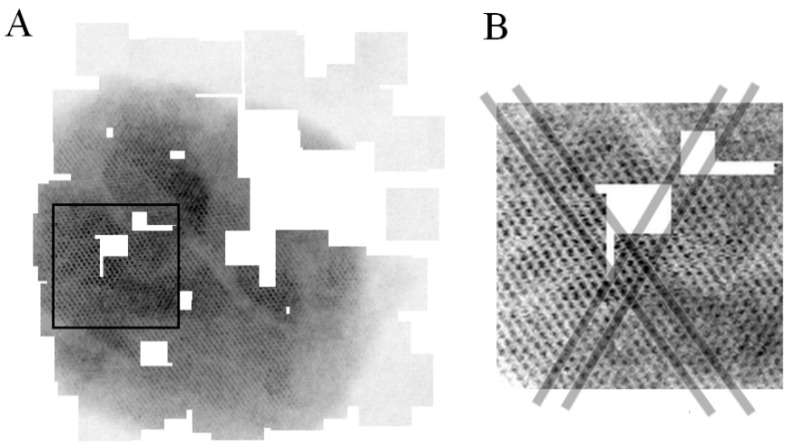
Block-based sparse imaging of an FeO nanoparticle at very high magnification. (**A**) Overlapping blocks (64 × 64 pixels) partially covering the FeO nanoparticle, white areas correspond to uncollected zones. (**B**) Zoom-in on a particular location of the nanoparticle (corresponding to the black box in (**A**) where the crystalline pattern is visible on several overlapping blocks. Thick grey lines drawn along the crystalline lattice highlight the alignment of the atom columns over several blocks.

In the work of Anderson et al., a similar strategy was attempted to perform non-linear scanning using the scanning coils of a scanning electron microscope and the authors pointed out the weak accuracy of the beam positioning in such non-trivial beam motions [27]. During the first tests, there was no apparent issue with the accuracy of the beam positioning on the JEOL 2200FS since the method reconstituted well the crossed line grating pattern (Figure 1). To further evaluate the accuracy of the beam positioning, the methodology was tested at much higher resolution with a second electron microscope, a CS-corrected JEOL 2100F. High-resolution images of the crystal structure of an iron oxide (FeO) nanoparticle [32] were obtained with that microscope (Figure 2). Overlapping blocks were collected so that positioning inaccuracies would be evidenced by the disruption of the crystalline lattice. Overlapping blocks are easily recognized and it is possible to focus on a region where the crystalline structure is visible over several overlapping blocks (black box in Figure 2A). Four thick grey lines have been drawn along two different axes of the crystal lattice passing through several blocks (Figure 2B). The atom columns are well aligned with the thick grey lines and no disruption of the crystalline lattice is observed. To further characterize the beam positioning, the distance between atom columns has been systematically measured and do not show visible discrepancy between collected blocks (Figure S1). The stability of the electron beam has been tested and verified with two different electron microscopes at different ranges of magnifications, validating the strategy to use Digiscan II and DM to drive the electron beam using the scanning coils.

### 3.2. Practical Implementation in 3D

The block-based sparse imaging and the reconstruction of the missing information using inpainting performed well on the crossed line grating replica. This sample has the advantage of being electron-resistant and possesses interesting features such as the Au/Pd grains and spherical latex beads, it is a sample of choice to develop the method for tomographic collection of sparse STEM images. The method relies on the acquisition of a tilt-series composed of sparse STEM images that are subsequently reconstructed using inpainting, then aligned by cross-correlation and eventually 3D reconstructed using WBP. The sparse 3D data are compared to a ground-truth reference made of fully-collected images.

#### 3.2.1. Collection of Sparse STEM Tilt-Series

**Figure 3:**
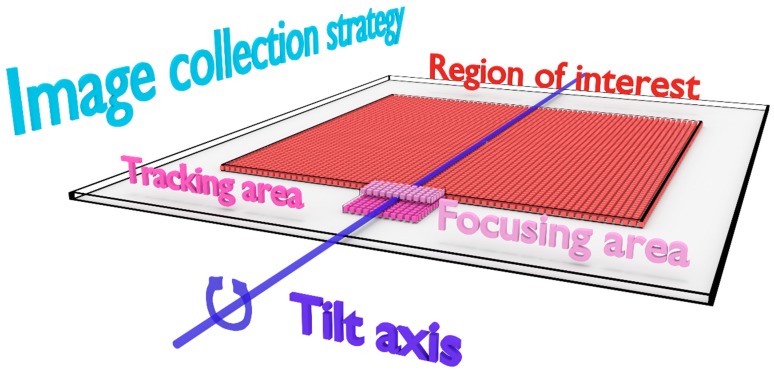
Strategy developed to collect sparse STEM images in a tomography workflow. The region of interest (in red) and the tracking and focusing areas (in dark and light pink respectively) are aligned with the tilt axis (in dark blue).

In a tomography workflow, there are two image processing steps that need to be performed accurately during the data collection: (i) the image registration to track the sample and (ii) the measurement of the focus value. Regarding the image registration step, the shifts between two consecutive tilts have to be computed to correct the sample drift. Regarding the focusing step, in STEM, it is usually performed by finding the most contrasted image among several images collected in a range of focus values. Sparse images might not have sufficient information in common to accurately perform image registration or contrast measurement. To ease the two image processing steps as mentioned above, they are performed on fully-collected images instead of sparse ones. Since the method aims to reduce the electron dose in the ROI, a second region next to the ROI and aligned with the tilt axis is used to acquire the fully-collected images to perform both focusing and tracking tasks (Figure 3). These areas are later on referred as focusing and tracking areas. The whole tomography workflow has been scripted and developed in DM. The script also codes for a graphical user interface to input the data collection parameters (Figure S2). The main steps of the script are the following:

- **Step 1—Focusing**: determination of the 0 nm focus value by successive acquisitions of fully-collected images on the focusing area in a focus range.
- **Step 2—Tracking**: acquisition of a fully-collected image on the tracking area and determination of the drift that occurred compared to the prior tilt angle by cross-correlation.
- **Step 3—Sparse data collection**: acquisition of the block-based sparse image on the region of interest.
- **Step 4—End of current tilt**: rotation to the next tilt angle and back to step 1.

#### 3.2.2. Validation of the Sparse STEM Tomography Workflow

**Figure 4:**
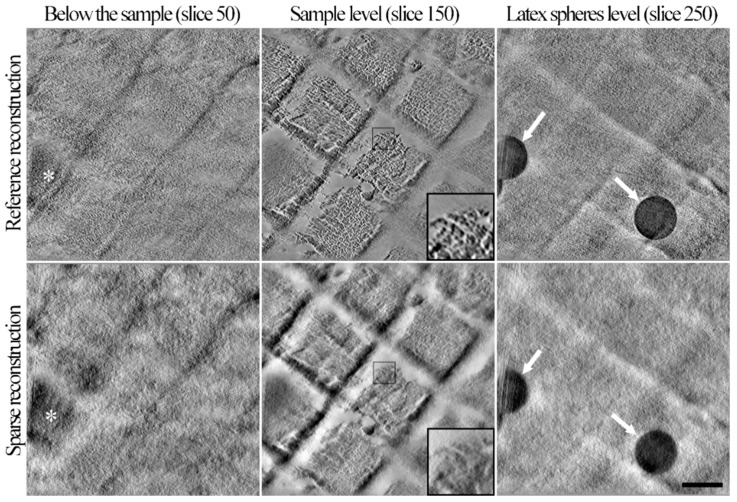
Comparison of the reference and sparse reconstructions. The reference reconstruction (**upper row**) has been computed using fully-collected images whereas the sparse reconstruction (**lower row**) has been computed using sparse images containing 6.25% of the pixels. For each reconstructed volume, three Z-slices were extracted: (i) below the sample (left column, Z-slices 50), (ii) at the level of the Au/Pd coating (center column, Z-slices 150) and (iii) above the sample at the level of the latex spheres (right column, Z-slices 250). The center area of Z-slices 150 (black boxes) has been zoomed-in to better visualize the details on the Au/Pd deposits. Reconstructions artifacts and latex spheres are indicated using white asterisks and white arrows, respectively. The scale bar is 250 nm. The six subfigures have the same scale.

For validation, the 3D reconstruction computed on sparse images has been compared with a reference volume. To this purpose, the sparse tomography workflow (6.25% downsampling) has been compared to a reference collection scheme (i.e., made of fully-collected images). Sparse images and fully-collected ones were consecutively collected at the same tilt-angle on the same ROI of a crossed line grating grid so that both images represent the object in the same orientation. After collection of the sparse data, missing pixels were first reconstructed using inpainting. Secondly, both tilt-series (i.e., sparse and reference ones) were aligned using the cross-correlation alignment parameters computed on the inpainted sparse images so that the quality of the alignment performed on inpainted data can be estimated. Finally, both sparse and reference 3D reconstructions were computed using WPB in Etomo (Figure 4). Z-slices 50 of the reconstructions (Figure 4, left column) do not contain any sample, however contrasted structures appear (Figure 4, white asterisks). These structures correspond to reconstruction artifacts indicating that the alignment could be improved. It can be noted that reconstruction artifacts in the sparse reconstruction are more visible than the ones in the reference reconstruction, most probably since sparse images originally contain fewer collected pixels (6.25% downsampling). At the level of the sample (Z-slices 150) the reference reconstruction is more contrasted and more detailed than the sparse one (Figure 4, center column). Individual Au/Pd deposits can be discriminated in the reference reconstruction whereas only global shapes of deposit clusters are visible in the sparse reconstruction (Figure 4, zoom-in). At the level of the latex spheres (Z-slices 250), similar reconstruction artifacts as the ones observed on Z-slices 50 are present (Figure 4, right column). Latex spheres (Figure 4, white arrows) are easily recognized in the sparse reconstruction and their roundness is conserved, though less defined than the ones in the reference reconstruction.

The 3D reconstruction computed from sparse images contains enough details to describe the overall structure of the carbon replica grid, the clusters of Au/Pd grains and latex spheres are visible. However, at such downsampling, the sparse reconstruction is not as detailed as the reference one, most probably because the very low amount of collected pixels cannot be compensated by the inpainting treatment. The presence of reconstruction artifacts showed that the cross-correlation alignment on inpainted sparse images could be improved. Potentially, better alignment could be computed if images of higher quality were used (i.e., if more pixels were collected). The carbon replica grid has been used as a test sample because it has several advantages. First, it is a thin sample that theoretically necessitates fewer projections to be accurately reconstructed in 3D compared to a thick sample. Secondly, its simple composition (low atomic variety) is theoretically better described using heavily downsampled sparse images compared to complex samples such as biological ones. The considerable downsampling (6.25%) used on the test sample authorizes some room to adapt the method to other types of sample if higher amounts of pixels need to be collected. These aspects are discussed in the following part that focuses on the application of the method on a 500 nm-thick section of resin-embedded *T. brucei* sample.

### 3.3. Application on a Biological Sample

**Figure 5:**
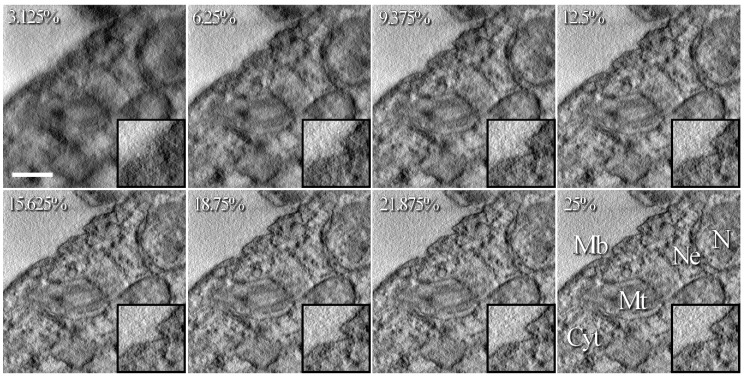
Comparison of 3D volumes reconstructed from sparse images collected at different downsampling values. The central Z-slice of each reconstruction is displayed. The value in the top left corner of each image corresponds to the downsampling value. Inserts in the bottom right corner are zoom-ins of the cell membrane. Several cellular structures are pointed out: the cytoplasm (Cyt), microtubules of the flagellum (Mt), the cell membrane (Mb), the nucleus (N) and the nuclear envelope (Ne). The scale bar is 400 nm. The eight subfigures have the same scale.

**Figure 6:**
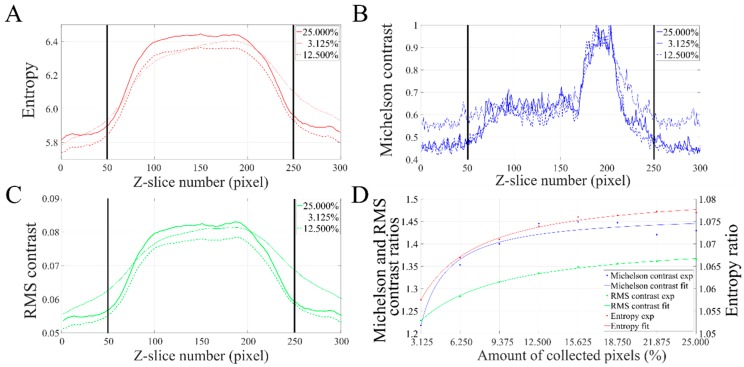
Image quality descriptors (IQD) values of sparse reconstructions depending on the amount of downsampling. (**A**–**C**) Plots of the *H*, *C_m_* and *C_rms_* descriptors computed at each Z slice of three reconstructions (3.125%, 12.5% and 25%), respectively. Thick vertical lines mark the location of the resin section in the reconstructed volumes (between Z-slices 50 and 250). (**D**) IQD ratios (experimental values and fitted curves) plotted by increasing order of downsampling.

The 500 nm-thick section of resin-embedded *T. brucei* represents the typical kind of sample that is studied in ET by life scientists. Eight tilt-series constituted of sparse images which downsampling ranged between 3.125% and 25% were collected on the 500 nm-thick resin section. Several downsampling values were tested to verify how low sparse imaging can be diminished while maintaining sufficient structural details. After data collection, tilt-series were inpainted, aligned using cross-correlation and reconstructed using WPB, giving rise to eight different volumes containing the ROI of the *T. brucei* section. The quality of the reconstructions has been assessed both visually (Figure 5) and using IQD (Figure 6). Displayed images represent the central slices of the reconstructions (Figure 5). The center of the ROI contains the flagellar pocket of a flagellum (Figure 5, Mt) and the nucleus of the cell is present in the top right corner (Figure 5, N). Visually, the amount of structural details in the reconstructions does not seem linear with the amount of collected pixels. At 3.125% downsampling, the reconstruction suffers important blurring and cellular structures are hardly recognizable. If the amount of collected pixels is doubled (6.25%) or tripled (9.375%), the increase in details is important and structures in the cell cytoplasm start to arise from the blurry background (Figure 5, Cyt). At 12.5% downsampling the structural information is more detailed, the membranes (Figure 5, Mb) are more continuous and the microtubule doublets (Figure 5, Mt) of the flagellum are better defined. Around the nucleus (Figure 5, N), the two membranes of the nuclear envelope (Figure 5, Ne) can be resolved. By eye, differences between reconstructions ranging from 15.625% to 25% downsampling are thin despite the important increase of collected pixels (e.g., there are 62.5% more pixels collected in the 25% downsampling compared to the 15.625% one). Plot profiles passing through known structures of the flagellar pocket of *T. brucei* were computed to better display the variation of pixel intensity in the various reconstructions (Figure S3).

To further characterize the reconstructions and to better describe thin differences that were not discernible by visual inspection of the volumes, IQD values were computed on individual Z-slices of all reconstructions. For the sake of clarity, because of the high number of reconstructions, only IQD values of some reconstructions (i.e., 3.125%, 12.5% and 25%) are presented (Figure 6A–C). Then, ratios between the average of IQD values of Z-slices passing through the resin section (i.e., between Z-slices 50 and 250) and the average of IQD values of Z-slices not passing through the resin section (i.e., the background, between Z-slices 1 and 50 and between Z-slices 250 and 300) have been computed and are used to evaluate the quality of the reconstructions (see Section 2.4). Ratios are presented and have been plotted by increasing order of downsampling value (Figure 6D).

For all reconstructions, IQD values at the level of the sample (i.e., between Z-slices 75 and 225) are greater than that of Z-slices where there is no sample (Figure 6A–C). Note that the high *C_m_* values around Z-slices 200 correspond to the presence of heavily contrasted structures in the reconstructions (Figure 6B). *C_m_* values being very sensitive to variations of pixels intensities because of the way it is computed, as displayed by its saw teeth shape. At 3.125% downsampling, when crossing from the resin section to the background (and vice versa), the slope of the IQD plots is not steep indicating that at very low amounts of collected pixels, the contrast of the object is poor (Figure 6A–C, continuous thin plots). At 12.5% downsampling, IQD plots have steeper slopes, allowing a clear discrimination between the object and the background (Figure 6A–C, dashed thick plots). At 25% downsampling, IQD values have similar behavior as that of 12.5% downsampling (Figure 6A–C, continuous thick plots). The ratio of IQD values has been computed to take into account the background noise so that reconstructions can be compared (Figure 6D). Ratios of IQD values greatly increase between 3.125% and 12.5% downsampling. At 3.125% downsampling, *C_m_*, *C_rms_* and *H* ratios are 1.2182, 1.2289 and 1.0575, respectively. At 12.5% downsampling, *C_m_*, *C_rms_* and *H* ratios are 1.4455, 1.3347 and 1.0740 respectively. At 25% downsampling, *C_m_*, *C_rms_* and *H* ratios reach 1.4296, 1.3650 and 1.0769 respectively. As can be seen with the fitted curves, above 12.5% downsampling, IQD ratios do not increase much and they start reaching a plateau around 25% downsampling. Based on the curve fitting, if all pixels were collected, ratios obtained for *C_m_*, *C_rms_* and *H* would be 1.50, 1.39 and 1.08 respectively. A table summarizing values of IQD ratios is available in Supplementary Information (Table S1). The three downsampling ratios presented in Figure 6A–C were chosen since they are good descriptors of the evolution of IQD values depending on the downsampling. The combination of (i) the three IQD measurements, (ii) the steepness of IQD plots at transitions between resin section and background and (iii) IQD ratios computed between the resin section and the background allows the characterization of thin differences that exist between the various reconstructions. The numerical IQD measurements (Figure 6) are in agreement with the visual inspection of the reconstructions (Figure 5).

## 4. Discussion

The aim of this work was to develop a tomography workflow to collect sparse images, only relying on the basic equipment of STEM electron microscopes to drive the electron beam (i.e., the scanning coils).

In the literature, scanning coils have previously been used to drive the electron beam in non-linear motions at sub-microsecond speeds. Anderson et al. used a dedicated system to send commands, receive detector signals and calibrate the mismatch between the desired beam position and the actual one since beam dynamics are important when high speed is achieved and when non-trivial motions are performed [27]. In the present study, the first step was to characterize how well the scanning coils of a JEOL 2200FS performed when they are commanded by Digiscan II. The first tests performed at relatively low magnification on the crossed line grating grid confirmed the correct positioning of the beam (Section 3.1.1). To push further the characterization, additional tests were performed on a CS-corrected JEOL 2100F at much higher magnification (Section 3.1.2). A good accuracy of the beam positioning was obtained and it confirmed that the strategy could be used on at least two different models of electron microscopes of the manufacturer JEOL. Somewhat, this high accuracy does not agree with what has previously been mentioned about beam dynamics [27]. It is possible that the scanning speed employed in the present study (between 1 µs and 8 µs per pixel) is slow enough not to introduce enough beam dynamics to produce a visible effect, even at high magnification. Furthermore, DM sets the block scan limit to 16 × 4 pixels and does not give the possibility to scan single pixels. This limit could exist to prevent beam dynamics from occurring. Such information could not be confirmed neither by people from Gatan USA nor Gatan France. Moreover, SEM studies are made at relatively low magnifications compared to TEM ones, hence large surfaces have to be scanned in SEM and the positioning of the beam might not be accurate when it is subjected to important deflections.

The collection time of a STEM image is defined by the number of collected pixels per line multiplied by the dwell time (time spent per pixel) and eventually by the number of lines in the image. When the beam reaches the end of a line, it is repositioned at the beginning of the next line (this step is called fly-back). Usually, the fly back time (about 150 to 250 µs) is relatively small compared to the time spent to collect the pixels of a single line (several thousands of µs). However, when scanning 16 × 4 pixel blocks using the scanning coils, a line is made of 16 pixels only and the fly-back command is called every 16 pixels. Eventually, the fly-back command is called so often that it contributes to a substantial part of the whole collection time. Hopefully, pixel blocks do not cover the whole ROI so the collection time stays reasonable. In practice, the whole acquisition of a tilt-series constituted of sparse images with 10% of the pixels, collected between −60° and +60° using 2° increments take about 220 min to complete. In the setups of Béché et al. and Vanrompay et al., a fast electromagnetic shutter was installed next to the condenser of the electron microscope so that they were able to blank the electron beam using custom sequences while the scanning coils performed standard linear acquisition [26,29]. In the setup of Anderson et al., the beam was scanned at very fast speeds (about 400 ns/pixel) using a custom scanning system [27]. Interestingly, these setups allowed the collection of sparse images at least as fast as standard linearly acquired images. Instead of collecting blocks, the acquisition of lines would be interesting to speed up the collection of sparse images using the scanning coils of the electron microscope as it is performed in Li et al. [28]. However, since lines cannot fill a ROI as efficiently as small blocks, with the exception maybe of Lissajous scans, similar downsampling values might not be reached.

Regarding the processing of the sparse images, several inpainting algorithms were tried and the one developed by Garcia [35,36] gave the best results in terms of visual quality. The inpainted sparse images do not have sufficient details to use fiducial-based [38] or landmark-based [41] alignment methods; hence, image registration was then performed by cross-correlation means. Reconstruction artifacts most probably originating from a perfectible alignment were observed on the crossed lined grating sample (Section 3.2.2) but not on the *T. brucei* sample (Section 3.3). Images of the crossed line grating sample had few latex spheres that had a strong contrast compared to the carbon where Au/Pd grains were deposited at the surface. Cross-correlation might fail in producing a good alignment on such images. On the contrary, images of the *T. brucei* sample were collected on a ROI that contained several cellular elements with strong contrast. The results show that the quality of the alignment is good enough to describe cellular structures in the reconstructed volume of a 500 nm-thick resin section. Testing other reconstruction algorithms such as iterative reconstruction methods or compressive-sensing approaches to verify which algorithm reconstructs the best such sparsely collected data is out of the scope of this work. Each reconstruction solution would need to be adapted to this specific kind of data.

If higher resolutions are required, cross-correlation solutions might not be sufficient to align the images with enough accuracy and other or new alignment methods should be employed, the main difficulty lying in the fact that the images of the tilt-series share very little amount of common information. One solution could be to generate an initial cross-correlation-based 3D reconstruction which alignment could be improved by iterative reconstruction algorithms that refine the alignment during the projection/back-projection comparison step, as described in previous studies [42,43]. Moreover, it seems necessary to design an algorithm that can discriminate between collected pixels (which intensity values have been measured experimentally) from inpainted ones (which intensity values have been estimated computationally). Experimental intensity values should have more weight in the reconstruction compared to estimated ones. Combining more robust image reconstruction algorithms such as wavelet- or shearlet-based inpainting and more accurate tilt-series alignment methods should help improving the resolution at levels comparable to resolutions achieved in classical ET.

Using the proposed approach; sparse STEM images containing only 15% of the pixels were collected on a 500 nm-thick resin section of *T. brucei*. The total electron dose received by the sample after the tilt-series collection was about 10 e^−^/Å^2^. The resulting 3D reconstruction contained enough structural details to recognize typical components present in the cytoplasm of eukaryotic cell. The method could be used both in material and life sciences to diminish the electron dose. In material sciences, similar strategies could be applied in 2D or in 3D to study in situ fragile samples such as nanowires [44] or Li-Ion batteries [45]. In life sciences, it could be used to study beam-sensitive cryo-samples in ET or in correlative experiments where the sample is exposed to different kinds of radiations. Correlative approaches are new powerful investigation methods and acquisition protocols limiting radiation damages could contribute to the development of correlative approaches yet to be proposed.

## Supplementary Materials

The following are available online at https://www.mdpi.com/1996-1944/12/14/2281/s1, Figure S1: Intra-block and inter-block distances between atom columns. (A) Image showing the location of blocks that have been collected on the FeO nanoparticle. Each block has a unique grey value to better discriminate them. The darker the block, the earlier (i.e., in time) it has been collected, hence the whiter the block, the later it has been collected. (B) Diagram presenting the locations where the distance between atom columns have been computed. Four thick grey lines highlight the precise locations. Distances have been measured on two different axes of the crystalline pattern not to miss a drift that would occur in one direction only, for more accurate error determination. (C) Close-up view showing the various superimposed blocks. It is possible to determine if two consecutive atom columns belong to the same collected block or if they belong to two different blocks. (D) Plot showing the measured distance between consecutive atom columns. Atom columns belonging to the same block (intra-block, blue and red points) and atom columns belonging to two different blocks (inter-block, blue and red circles) have been plotted separately. Blue and red plots represent values measured on axis1 and axis2 respectively. Mean distance values for axis1 (intra-block), axis1 (inter-block), axis2 (intra-block) and axis2 (inter-block) are 5.47 (std: 0.96), 6.22 (std: 1.09), 6.24 (std: 1.223), 5.64 (std: 0.86) pixels respectively. Intra-block values show variations originating most probably from noise and crystalline defects in the nanoparticle. Inter-block measurements have similar values, demonstrating that the beam positioning does not introduce disruption of the crystalline lattice, proving that the method is accurate enough to perform sparse imaging using pixel blocks. Figure S2: Graphical interface of the Digital Micrograph script to perform sparse STEM tomography. Standard parameters such as the microscope magnification, the dwell time, the angular range and the tilt-step are set up in the interface. Parameters specific to the sparse imaging are present: scan (block) size (e.g., 16 × 4 px), the reconstructed image size (e.g., 2000 px) and the number of collected scans (blocks) (e.g., 512). The focus value parameter serves as an initial value around which the script will search for the 0 nm defocus value. Figure S3: Intensity profiles of the same ROI in 3D volumes reconstructed from variously downsampled sparse STEM images. The profiles have taken through the flagellum of *T. brucei* at the position indicated by the pointed yellow line on the tomographic slice. The height of the intensity profiles does not inform about the intensity values, they have been placed on top of each other for the sake of clarity. The intensity profiles cross several structures of the cell, the cytoplasm (Cyt), the flagellar pocket membrane (FPM), the flagellar pocket space (FPS), the flagellar membrane (FM), the transition zone space (TZS), the microtubule doublets of the axoneme (Mt) and the axonemal space (AS). If downsampling is lower than about 10%, intensity variation from one structure to the other is hardly recognizable. When greater amounts of pixels are collected, strong intensity variations allow to discriminate the structures. Above 15% downsampling the intensity profiles are very similar one another. Table S1: Table summarizing values of IQD ratios for three downsampling (3.125%, 12.5% and 25%) and for the estimated 100% corresponding to no downsampling. The first row contains the downsampling values. The last column represents the values extrapolated from the fitted curves. Values in between brackets represent the ratio with respect to the extrapolated values.
