# Supplementary material for: Tomographic Collection of Block-Based Sparse STEM Images: Practical Implementation and Impact on the Quality of the 3D Reconstructed Volume
Source: Materials (Basel). 2019 Jul 16;12(14):2281. doi: 10.3390/ma12142281 (PMC6679239; doi:10.3390/ma12142281)
Supplement: Supplementary file 1 [file materials-12-02281-s001.pdf]

*materials*

*Supplementary Materials*

# Tomographic Collection of Block-Based Sparse STEM images: Practical Implementation and Impact on The Quality of the 3D Reconstructed Volume

**Sylvain Trépout**

Institut Curie, Inserm U1196, CNRS UMR 9187, Université Paris Sud, Centre Universitaire, Bât. 110-112, 91405 Orsay Cedex, France
Correspondence: sylvain.trepout@curie.fr; Tel.: +33-169-86-30-81

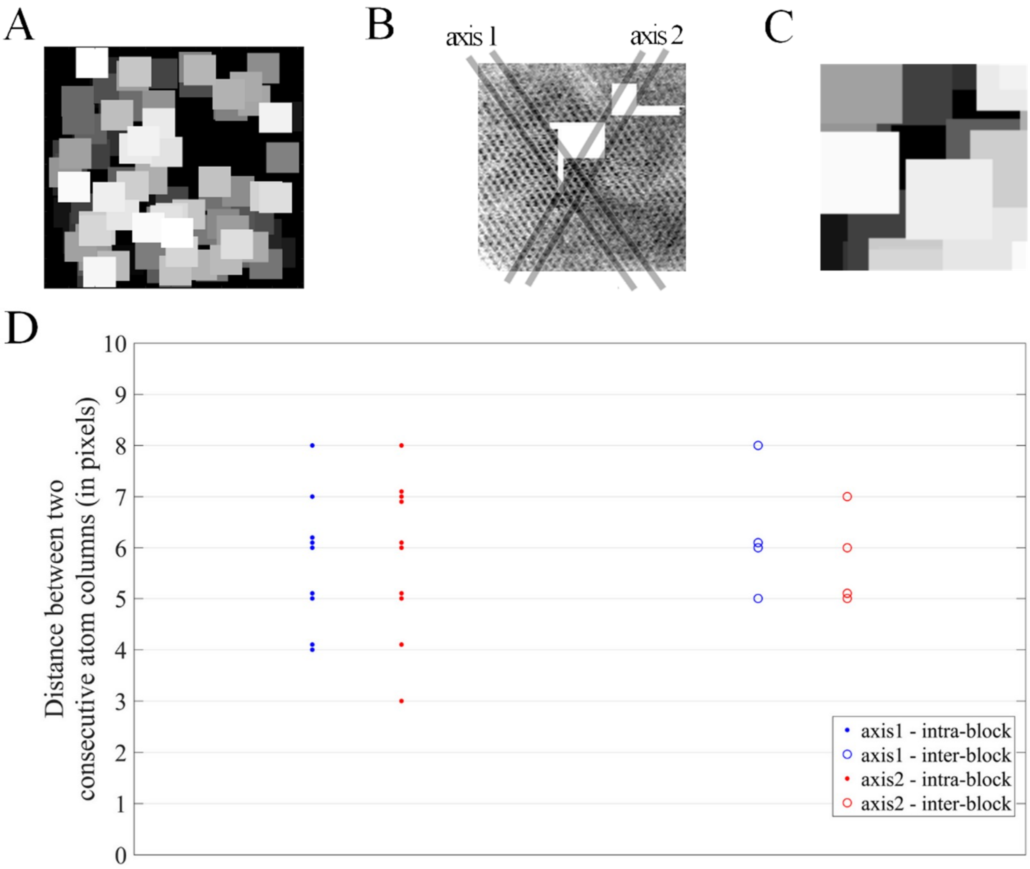


**Figure S1.** Intra-block and inter-block distances between atom columns. (A) Image showing the location of blocks that have been collected on the FeO nanoparticle. Each block has a unique grey value to better discriminate them. The darker the block, the earlier (i.e. in time) it has been collected, hence the whiter the block, the later it has been collected. (B) Diagram presenting the locations where the distances between atom columns have been computed. Four thick grey lines highlight the precise locations. Distances have been measured on two different axes of the crystalline pattern not to miss a drift that would occur in one direction only, for more accurate error determination. (C) Close-up view showing the various superimposed blocks. It is possible to determine if two consecutive atom columns belong to the same collected block or if they belong to two different blocks. (D) Plot showing the measured distance between consecutive atom columns. Atom columns belonging to the same block (intra-block, blue and red points) and atom columns belonging to two different blocks (inter-block, blue and red circles) have been plotted separately. Blue and red plots represent values measured on axis1 and axis2 respectively. Mean distance values for axis1 (intra-block), axis1 (inter-block), axis2 (intra-block) and axis2 (inter-block) are 5.47 (std: 0.96), 6.22 (std: 1.09), 6.24 (std: 1.223), 5.64 (std: 0.86) pixels respectively. Intra-block values show variations originating most probably from noise and crystalline defects in the nanoparticle. Inter-block measurements have similar values, demonstrating that the beam positioning does not introduce disruption of the crystalline lattice, proving that the method is accurate enough to perform sparse imaging using pixel blocks.

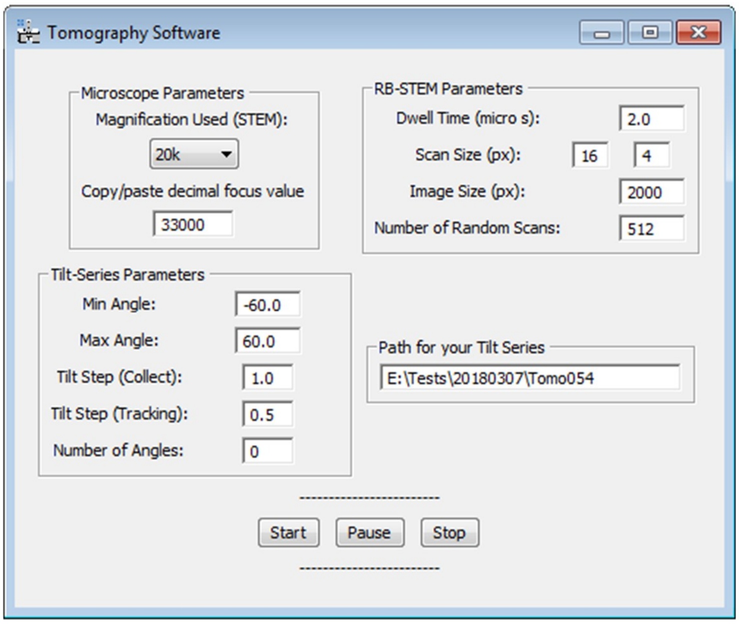


**Figure S2.** Graphical interface of the Digital Micrograph script to perform sparse STEM tomography. Standard parameters such as the microscope magnification, the dwell time, the angular range and the tilt-step are set up in the interface. Parameters specific to the sparse imaging are present: scan (block) size (e.g., 16 × 4 px), the reconstructed image size (e.g., 2000 px) and the number of collected scans (blocks) (e.g., 512). The focus value parameter serves as an initial value around which the script will search for the 0 nm defocus value.

materials

MDPI

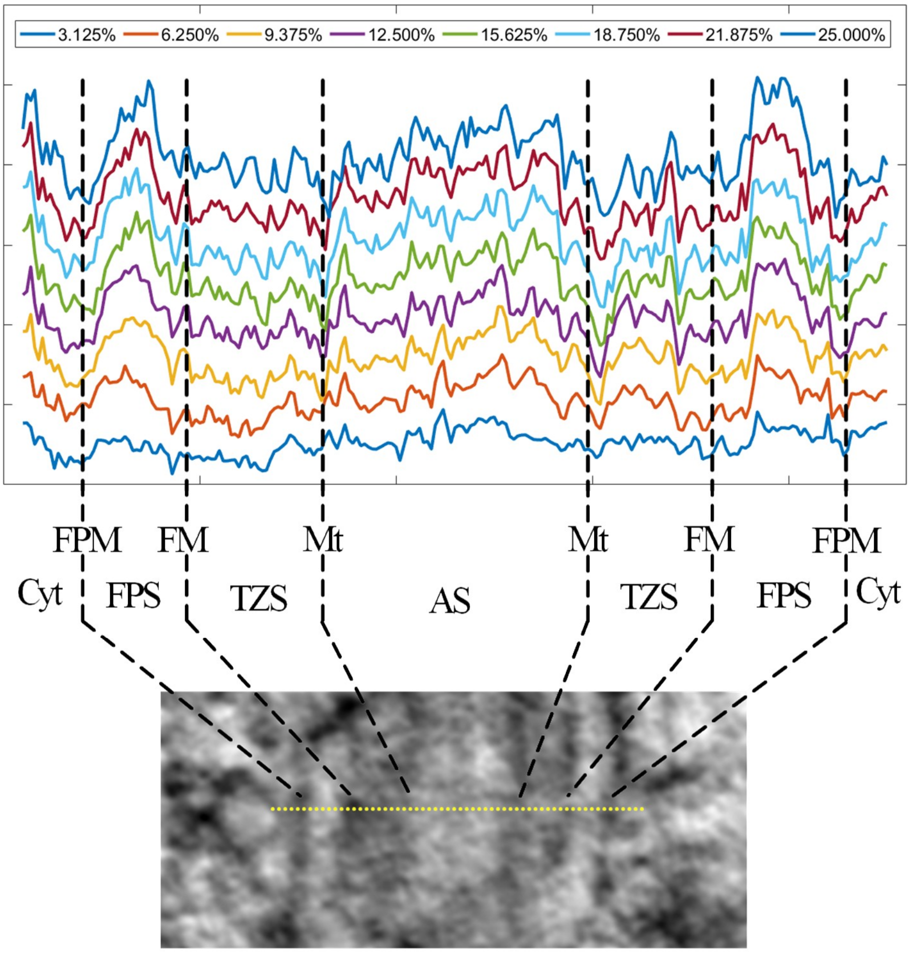


**Figure S3.** Intensity profiles of the same ROI in 3D volumes reconstructed from variously downsampled sparse STEM images. The profiles have taken through the flagellum of *T. brucei* at the position indicated by the pointed yellow line on the tomographic slice. The height of the intensity profiles does not inform about the intensity values, they have been placed on top of each other for the sake of clarity. The intensity profiles cross several structures of the cell, the cytoplasm (Cyt), the flagellar pocket membrane (FPM), the flagellar pocket space (FPS), the flagellar membrane (FM), the transition zone space (TZS), the microtubule doublets of the axoneme (Mt) and the axonemal space (AS). If downsampling is lower than about 10%, intensity variation from one structure to the other is hardly recognizable. When greater amounts of pixels are collected, strong intensity variations allow to discriminate the structures. Above 15% downsampling the intensity profiles are very similar one another.

**Table S1.** Table summarizing values of IQD ratios for three downsampling (3.125%, 12.5% and 25%) and for the estimated 100% corresponding to no downsampling. The first row gives the downsampling values. The last column represents the values extrapolated from the fitted curves. Values in between brackets represent the ratio with respect to the extrapolated values.

| Downsampling | 3.125% | 12.5% | 25% | 100% (fit estimation) |
|---|---|---|---|---|
| $C_m$ | 1.2182 (0.81) | 1.4455 (0.96) | 1.4296 (0.95) | 1.5 |
| $C_{rms}$ | 1.2289 (0.88) | 1.3347 (0.96) | 1.3650 (0.98) | 1.39 |
| $H$ | 1.0575 (0.98) | 1.0740 (0.99) | 1.0769 (1) | 1.08 |

© 2019 by the authors. Licensee MDPI, Basel, Switzerland. This article is an open access article distributed under the terms and conditions of the Creative Commons Attribution (CC BY) license (http://creativecommons.org/licenses/by/4.0/).
